# Contributions to Practical Surgery

**Published:** 1873-09

**Authors:** 


*Contributions to Practical Surgery.* By George W. Norris, M. D., Philadelphia: Lindsay & Blakeston, 1873. Buffalo: T. Butler & Son.

A large number of the articles in this work have previously appeared in print, and some have attracted considerable attention both in this country and abroad. They are now for the first time brought together in book form and the result is one of the best contributions to surgery which it has been our lot to peruse for some time. The number of subjects treated in the work are not large, but those presented are handled in so complete a manner as to leave nothing to be desired. The paper on Compound Fractures, is entirely new, and many additions have been made to the other papers embracing clinical histories drawn from hospital practice. The most notable addition is that made on the occurrence of false joints. The first paper is upon the occurrence of non-union after fracture, and embraces one hundred and eleven pages. It is a full and complete review of the whole subject, embracing a consideration of the various theories concerning its cause, and the different methods of treatment which have been used. From this article it will be seen that non-union after fracture is most frequent in the humerus and femur, that the mortality after operations for cure follow the same law as after amputations, viz., that the danger increases with the size of the limb, and the nearness of the operation to the trunk. Failures are found to be most frequent in the humerus, and in middle-aged and elderly than in younger subjects. The author also arrives at the conclusion which will we think meet the views of all that the seton and its modifications is safer and speedier than resection or the caustic.

The treatment of Deformities following Unsuccessfully Treated Fracture is next considered. The views advanced are such as will meet with the approval of the profession; an interesting account of several operations are given in this connection.

The paper on Compound Fractures, is to us one of the most interesting, of the many interesting articles in the book. The author's long service in the Pennsylvania Hospital have given him an experience which will entitle him to speak with authority. The paper discusses in a complete manner the pracical treatment of compound fractures, and will be found in most particulars to agree with generally accepted views. We can hardly agree with the writer in leaving all splinters of bone which are adherent to the soft parts to become spontaneously detached. We have been in the habit of removing all splinters whether adherent or not, when there was no probability of their becoming again united to the shaft, and have yet to see any serious result either in the length of the limb or the union of the fracture. On the contrary we have in several instances seen both limb and life lost from the neglect to remove these splinters. In this connection it may be well to notice that although the author has not had a large experience in removing the ends of bones to facilitate the reduction of a compound fracture he speaks with favor of the operation. In this he meets with our highest approval. Although it has been, and is doubtless, practiced needlessly in many cases, yet we can call to mind many instances wherein much trouble from necrosis etc., would have been saved, and a better result secured in the end, had a resection been made at the first dressing.

Statistics of the cases of amputation performed at the Pennsylvania Hospital from Jan. 1850 to Jan. 1860, and a paper giving the statistics of the mortality following the ligature of arteries comprise the next two articles. An account of a case of Varicose Aneurism at the bend of the arm, is the concluding article of the book. The book is printed on smooth heavy paper and is handsomely bound. We have been very much interes ed in reading the various articles, and recommend it to our readers as a work from which much valuable information can be obtained.

